# The Membrane-Associated Transient Receptor Potential Vanilloid Channel Is the Central Heat Shock Receptor Controlling the Cellular Heat Shock Response in Epithelial Cells

**DOI:** 10.1371/journal.pone.0057149

**Published:** 2013-02-27

**Authors:** Zohar Bromberg, Pierre Goloubinoff, Younousse Saidi, Yoram George Weiss

**Affiliations:** 1 Dept. of Anesthesiology and Critical Care Medicine and the Goldyne Savad Institute of Gene Therapy, Hadassah-Hebrew University School of Medicine, Jerusalem, Israel; 2 Dept. of Plant Molecular Biology, University of Lausanne, Lausanne, Switzerland; 3 Dept. of Anesthesiology and Critical Care Medicine, University of Pennsylvania School of Medicine, Philadelphia, Pennsylvania, United States of America; St. Georges University of London, United Kingdom

## Abstract

The heat shock response (HSR) is a highly conserved molecular response to various types of stresses, including heat shock, during which heat-shock proteins (Hsps) are produced to prevent and repair damages in labile proteins and membranes. In cells, protein unfolding in the cytoplasm is thought to directly enable the activation of the heat shock factor 1 (HSF-1), however, recent work supports the activation of the HSR via an increase in the fluidity of specific membrane domains, leading to activation of heat-shock genes. Our findings support the existence of a plasma membrane-dependent mechanism of HSF-1 activation in animal cells, which is initiated by a membrane-associated transient receptor potential vanilloid receptor (TRPV). We found in various non-cancerous and cancerous mammalian epithelial cells that the TRPV1 agonists, capsaicin and resiniferatoxin (RTX), upregulated the accumulation of Hsp70, Hsp90 and Hsp27 and Hsp70 and Hsp90 respectively, while the TRPV1 antagonists, capsazepine and AMG-9810, attenuated the accumulation of Hsp70, Hsp90 and Hsp27 and Hsp70, Hsp90, respectively. Capsaicin was also shown to activate HSF-1. These findings suggest that heat-sensing and signaling in mammalian cells is dependent on TRPV channels in the plasma membrane. Thus, TRPV channels may be important drug targets to inhibit or restore the cellular stress response in diseases with defective cellular proteins, such as cancer, inflammation and aging.

## Introduction

The heat shock response (HSR), which is induced upon exposure of living cells to acute or subacute stressors, is characterized by the expression of a group of phylogenetically-conserved intracellular heat shock proteins (HSPs), which have a protective effect against injury, especially of proteins [1]. Among the most massively expressed HSPs are molecular chaperones that mediate conformational changes in proteins and prevent protein misfolding. Some heat-induced chaperones, such as Hsp70, can hydrolyze ATP to unfold toxic protein aggregates into natively refoldable polypeptides and promote the degradation of toxic misfolded proteins by the proteasome [2]–[5]. Further, HSP chaperones modulate apoptosis, NF-κB signaling, cell division, and other pathways associated with the cellular response to stress, resulting in cytoprotection from various environmental stresses, heat shock, and infection-induced pro-inflammatory pathways [1], [2], [6]–[9]. Hsp70 is a core element of the chaperone network in the cell that controls stress signaling, protein trafficking and the onset of damage-preventing and repairing mechanisms [3], [10]. Hsp70 functions in collaboration with chaperones and co-chaperones such as Hsp90, Hsp110, Hsp27, Hsp40, etc. [1]–[3]. Altered levels of HSP chaperones are often observed in inflammation, as in severe sepsis [11]. The HSR also provides cytoprotection from chemical aggressors, such as chemotherapy [12)]. Hence, cancer cells that constitutively up-regulate the expression of HSPs, Hsp70 and Hsp90 in particular, are often resistant to heat as well as to chemotherapeutic treatments [12]. Reciprocally, inhibition of Hsp90 using specific inhibitors such as geldanamycin or radicicol, or reducing Hsp70 expression using siRNA, leads to growth arrest and cell death [13]–[16].

Heat shock factors (HSFs), in particular HSF-1, control the transcription of HSPs [4]. In the absence of stress, inactive HSF-1 monomers are hypo-phosphorylated and are apparently associated in the cytoplasm with Hsp70 and Hsp90 [17]. One mechanism by which diverse stress stimuli, heat-shock or toxic chemicals in particular, are proposed to induce the HSR, is the presumed stress-induced formation of misfolded proteins in the cytoplasm, which would in turn titrate Hsp70 and Hsp90 chaperones away from their stable inhibitory association with HSF-1, thereby, presumably, resulting in HSF-1 activation by way of hyper-phosphorylation, trimerization and translocation to the nucleus [4], [18]–[20]. However, this mechanism fails to explain situations where HSP levels are increased in the presence of non-denaturing chemicals and without heat under physiological conditions, where protein aggregates are unlikely to form in the cell. Thus, a strong HSR may already develop in response to a very mild temperature increase or in the presence of membrane-interfering compounds such as arimoclomol, benzyl alcohol, NG-094, BGP-15 [21]–[26]. A different mechanism for heat-sensing and for activation by heat or chemicals of the HSR has been demonstrated whereby activation of the HSR occurs via increase in the fluidity of specific membrane microdomains, leading to activation of heat-shock genes [22]. This activation of the HSR occurs, not only by heat, but may also happen in response to chemicals, such as bimoclomol sterol glycoside [27], acetyl salicylic acid or arachidonic acid [25]. The activation occurs through subsequent secondary messengers, such as calmodulin-binging domains, which are likely to associate with entering Ca-ions and specific calmodulins in the cytoplasm [27], sterol glycoside [27] or arachidonic acid [25], thus providing cells with a means to mediate a highly specific Heat Shock-like signal without temperature changes.

Here, we provide new supportive evidence to the seminal findings that binding of HSF-1 to HSE can be activated by the entry of extracellular Ca^2+^ also in mammalian cells [28]. This work identifies specific Ca^2+^-channels as components of a central cellular thermosensory apparatus in mammalian cells. We show that mammalian epithelial cells contain specific Ca^2+^-channels, the membrane-dependent transient receptor potential vanilloid (TRPV) that can respond primarily to a temperature rise and also to chemicals such as capsaicin, by sending a Ca^2+^-entry dependent signal to activate HSF-1 and develop a cellular HSR. We found in various non-cancerous and cancerous mammalian epithelial cells that the TRPV1 agonists, capsaicin and resiniferatoxin (RTX) [29], upregulated the accumulation of Hsp70, Hsp90 and Hsp27 and Hsp70 and Hsp90, respectively, while the TRPV1 antagonists, capsazepine, and AMG-9810 [30] attenuated the accumulation of Hsp70, Hsp90 and Hsp27 and Hsp70, Hsp90, respectively. Capsaicin was also shown to activate HSF-1. TRPV1 siRNA, capsazepine, or AMG-9810, or extracellular EGTA all prevented the heat-induced accumulation of Hsp70 and Hsp90, suggesting that in mammalian cells, heat-sensing and cellular stress signaling in general depend on specific membrane-dependent transient receptor potential vanilloid (TRPV) calcium channel-like receptors. Together, our findings suggest that heat or specific chemicals can change the plasma membrane state [22], thus triggering the transient opening of calcium channel-like receptors (TRPVs), which in turn send a specific signal to generate a cellular stress response, under conditions that are not necessarily dependent on stress damages in proteins, in particular the formation of intra-cellular protein aggregates.

## Results

### Capsaicin induces, and capsazepine inhibits HSP expression in non-neuronal epithelial cells

**Figure 1:**
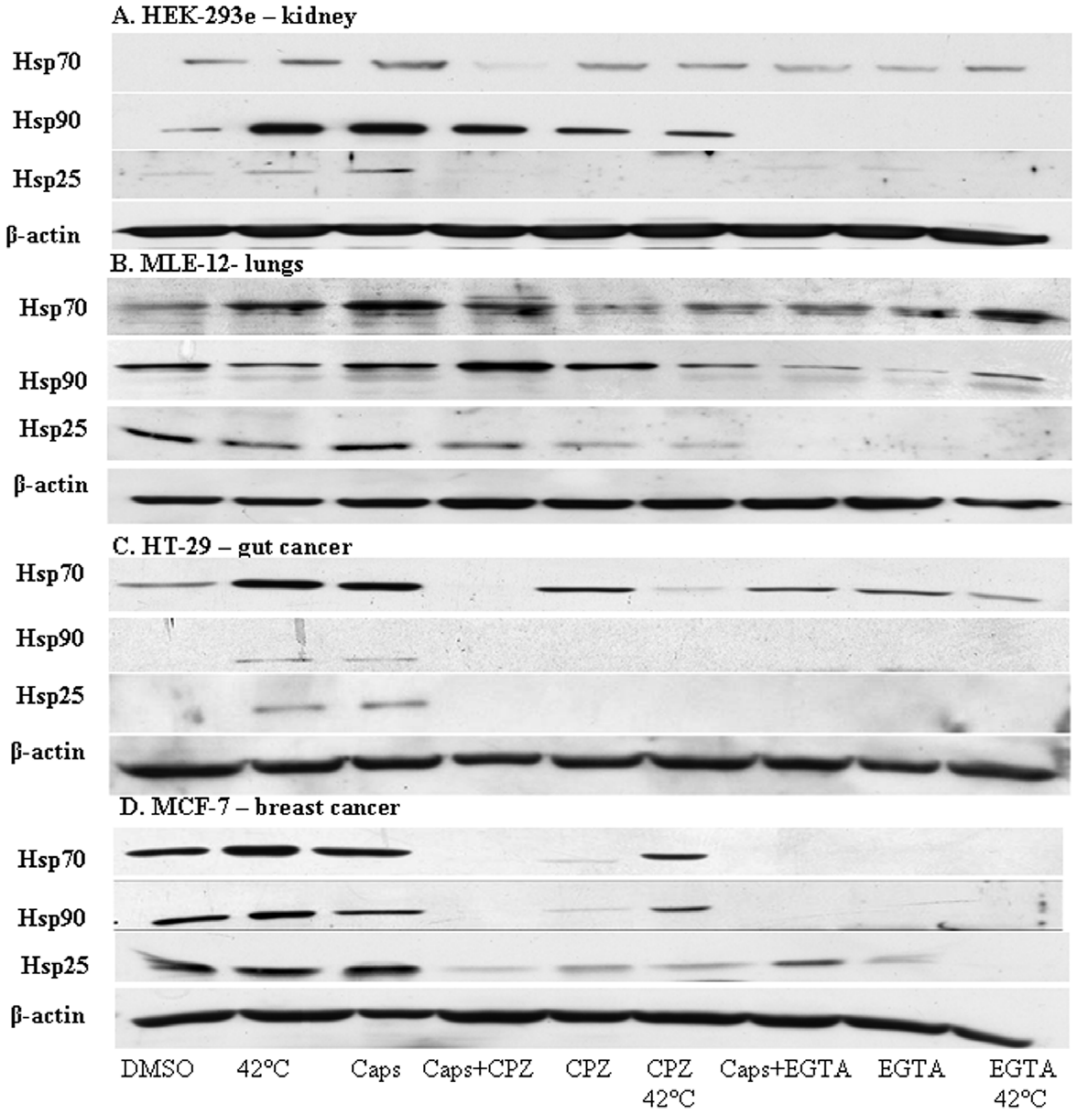
Capsaicin and capsazepine effects on heat shock protein expression in HEK293e, MLE-12, MCF-7 and HT-29 cells. Representative immunoblot analysis of Hsp70, Hsp90 and Hsp27 abundances. Hsp70, Hsp90 and Hsp27 were detected in HEK293e (Panel A), MLE-12 cells (Panel B), HT-29 (Panel C) and MCF-7 (Panel D). Cells in culture were incubated with 0.01% DMSO, 32 µM of capsaicin (Caps) for 1 hr, 32 µM of capsaicin (Caps) for 1 hr with the addition of 100 µM capsazepine (CPZ) or 100 µM capsazepine alone at 37°C. Cells were treated with either heat shock at 42°C for 3 hrs, heat shock at 42°C for 3 hrs with the addition of 100 µM capsazepine. Cells were treated with 32 µM of capsaicin with the addition of 5 mM EGTA, 5 mM EGTA alone at 37°C or heat shock at 42°C for 3 hrs with the addition of 5 mM EGTA. 15 µg of total protein/lane were separated on 9% SDS-PAGE gel.

**Figure 2:**
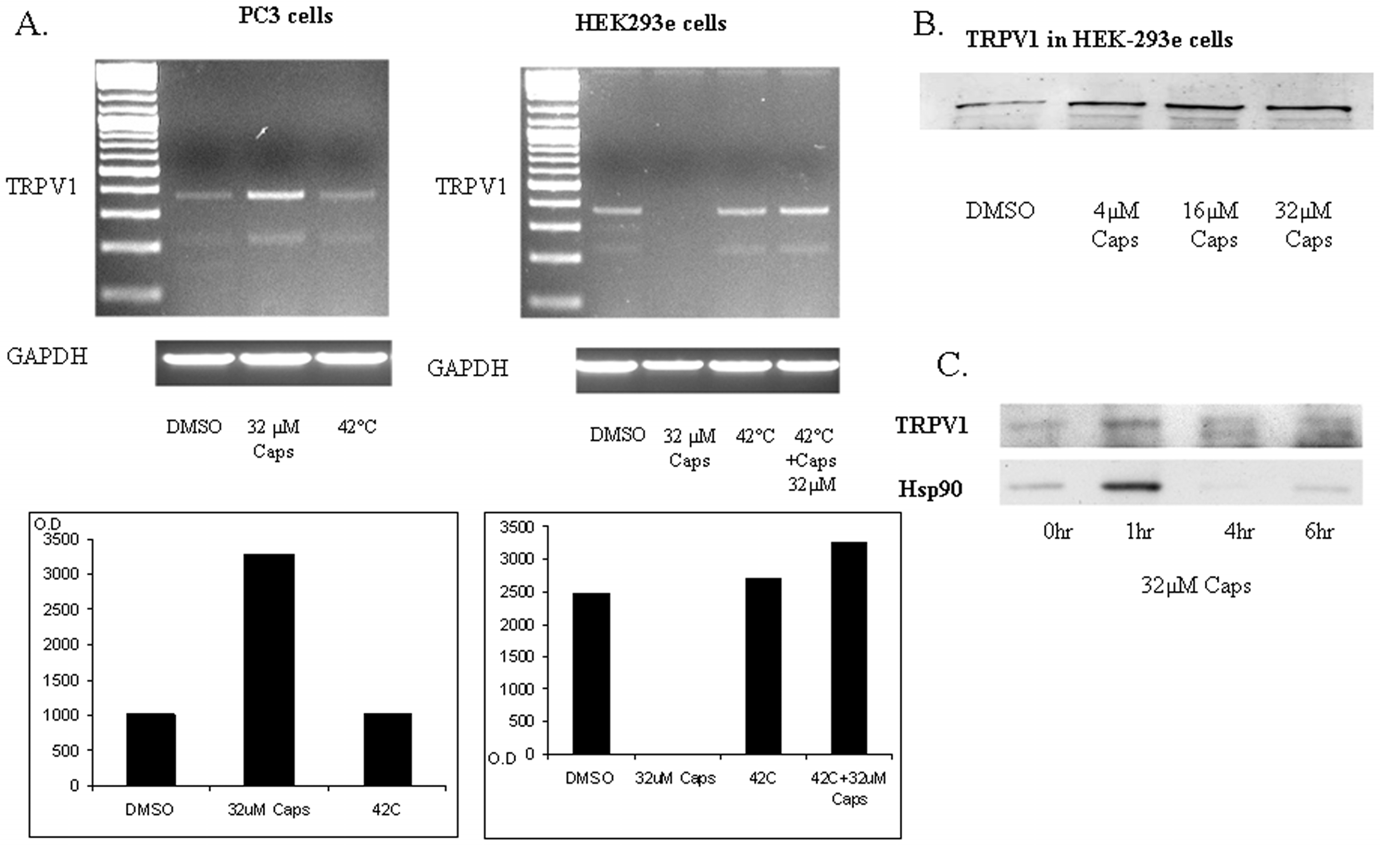
TRPV1 mRNA and protein abundances in HEK-293e cells. A. Semi-quantitative RT-PCR for TRPV1. Upper left panel: mRNA levels from PC3 prostate cancer cells: non treated cells, cells treated with 32 µM capsaicin for 1 hr and cells treated with heat shock at 42°C for 3 hrs. Lower right panel: mRNA levels of GAPDH as well as densitometric. Upper right panel: mRNA levels from HEK-293e cells: non treated cells, cells treated with 32 µM capsaicin for 1 hr (Caps), cells treated with heat shock at 42°C for 3 hrs, and cells treated with heat shock at 42°C for 3 hrs with the addition of 32 µM capsaicin. Lower right Panel: mRNA levels of GAPDH as well as densitometry. B. Representative immunoblot analysis of TRPV1 abundance. TRPV1 was detected in HEK-293e cells. Cells in culture were incubated with 0.01% DMSO or treated with 4 µM, 16 µM or 32 µM of capsaicin for 1 hr. C. Representative immunoblot analysis of TRPV1 and Hsp90 abundances in HEK-293e cells. Cells were treated with 32 µM capsaicin for 0 hr, 1 hr, 4 hrs and 6 hrs.

Western blot analysis showed that when kidney cells (HEK-293e found to express TRPV1), alveolar epithelial cells (MLE-12), carcinoma breast cancer cell line (MCF-7) or colon cancer cells (HT-29) are treated with the TRPV1 agonist capsaicin, they accumulate Hsp70, Hsp90 and Hsp27 proteins (**Fig. 1**). Capsazepine, a specific antagonist of capsaicin [28], [29], abolished the heat-induced HSR (**Fig. 1**). Capsaicin and capsazepine were found to respectively activate, or inhibit the HSR. Similarly to capsazepine, treatment with the strong calcium chelator EGTA abolished HSP overexpression by capsaicin or by heat (**Fig. 1**). Furthermore, exposure of HEK-293e cells to 32 µM capsaicin, at different times (0, 1 hr, 4 hrs, and 6 hrs) demonstrated significantly elevated HSP levels after 1 hr (**Fig.2C**).

**Figure 3:**
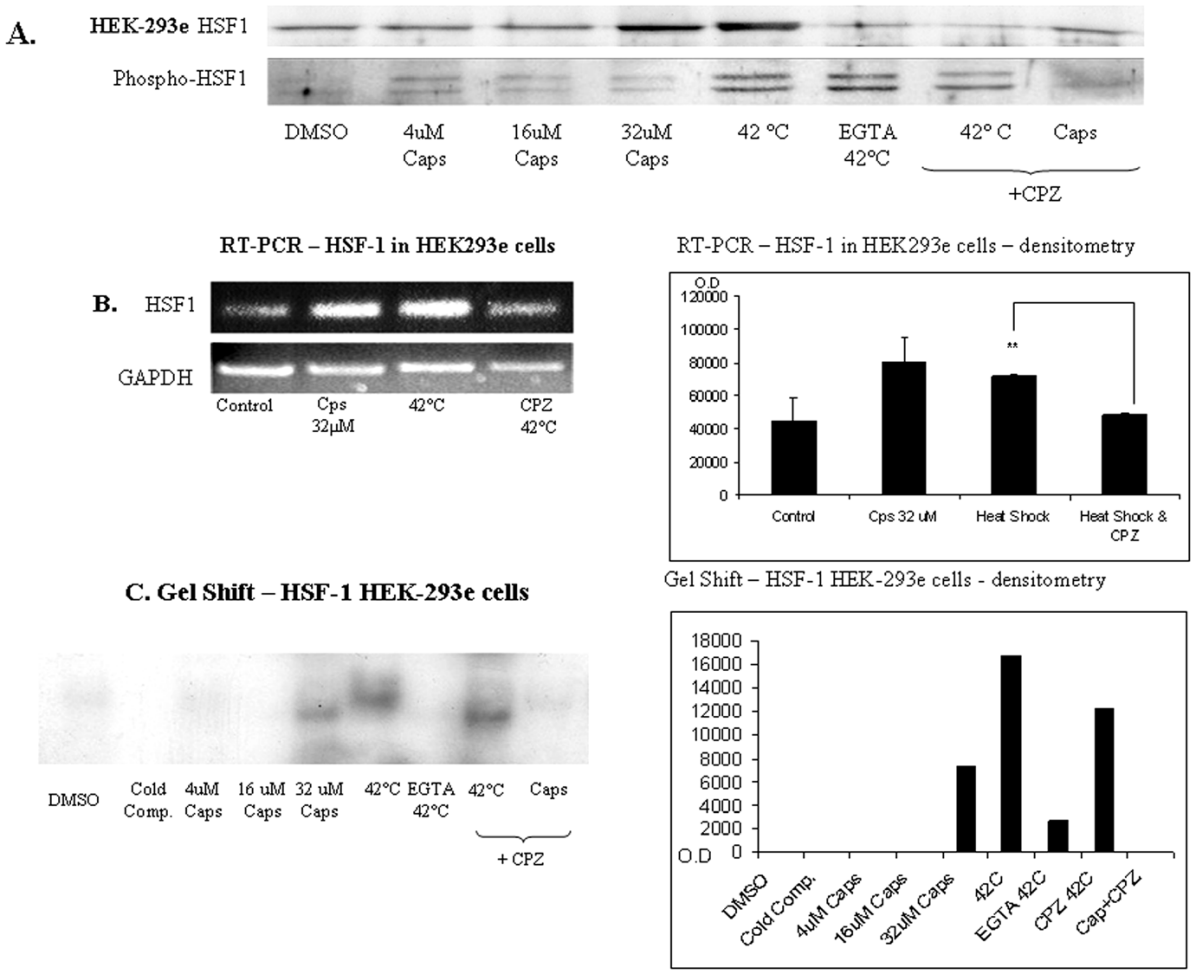
Capsazepine inhibits HSF-1 expression and nuclear translocation. A. Representative immunoblot analysis of nuclear HSF-1 and phospho-HSF-1 abundances. Intra-nuclear HSF-1 and Phospho-HSF-1 were detected in HEK-293e cells. Cells were incubated with 4 µM, 16 µM or 32 µM of capsaicin for 1 hr. HEK-293e cells were treated as indicated with heat shock at 42°C for 3 hrs, heat shock at 42°C for 3 hrs with the addition of 5 mM EGTA, heat shock at 42°C for 3 hrs with the addition of 100 µM capsazepine or 100 µM capsazepine with the addition of capsaicin. B. Semi-quantitative RT-PCR for HSF-1. Upper panel: mRNA levels from non treated HEK293e cells (control), cells treated with 32 µM capsaicin for 1 hr (Caps), cells treated with heat shock at 42°C for 3 hrs, cells treated with heat shock at 42°C for 3 hrs with the addition of 100 µM capsazepine. Lower Panels: GAPDH mRNA levels from HEK293e cells as indicated above as well as densitometric analysis (Right panel). C. Electrophoretic mobility shift assay for HSF-1 DNA binding activity. Nuclear protein isolated from HEK-293e cells treated with 4 µM, 16 µM or 32 µM of capsaicin for 1 hr. Cells treated as indicated with heat shock at 42°C for 3 hrs, heat shock at 42°C for 3 hrs with the addition of 5 mM EGTA, heat shock at 42°C for 3 hrs with the addition of 100 µM capsazepine or 100 µM capsazepine with the addition of capsaicin. 10 µg of nuclear protein incubated for 20 min at room temperature with a ^32^P-labeled double-stranded DNA oligonucleotide containing a consensus –HSF-1 binding site. Right panel indicates densitometric analysis.

A 3-hour heat-shock at 42°C or an isothermal treatment with capsaicin, both induced the accumulation of HSPs, which was directly associated with an increase of HSF-1 and Phospho-HSF-1 abundance and, expectedly, with HSF-1 activation, as determined also by DNA-shift binding activity in HEK-293e cells (**Fig. 3**). Confirming the isothermal induction we show that in cells harboring TRPV1, either heat-shock or treatment with capsaicin induces an increase of HSF-1 and Phospho-HSF-1 abundances, a finding confirmed by semi quantitative PCR depicting an increase of HSF-1 mRNA (**Fig. 3**). The addition of capsazepine prior to HS impaired the heat-dependent induction of HSF-1 (**Fig. 3B**). Capsazepine-suppression of heat-induced HSP accumulation was directly associated with the inhibition of HSF-1 abundance and with its reduced DNA binding activity (**Fig. 3C**). Treatment of cells with both capsaicin and its competitive inhibitor capsazepine, resulted in a partial inhibition of the capsaicin-induced HSR (**Fig. 1** **&** **3**).

**Figure 4:**
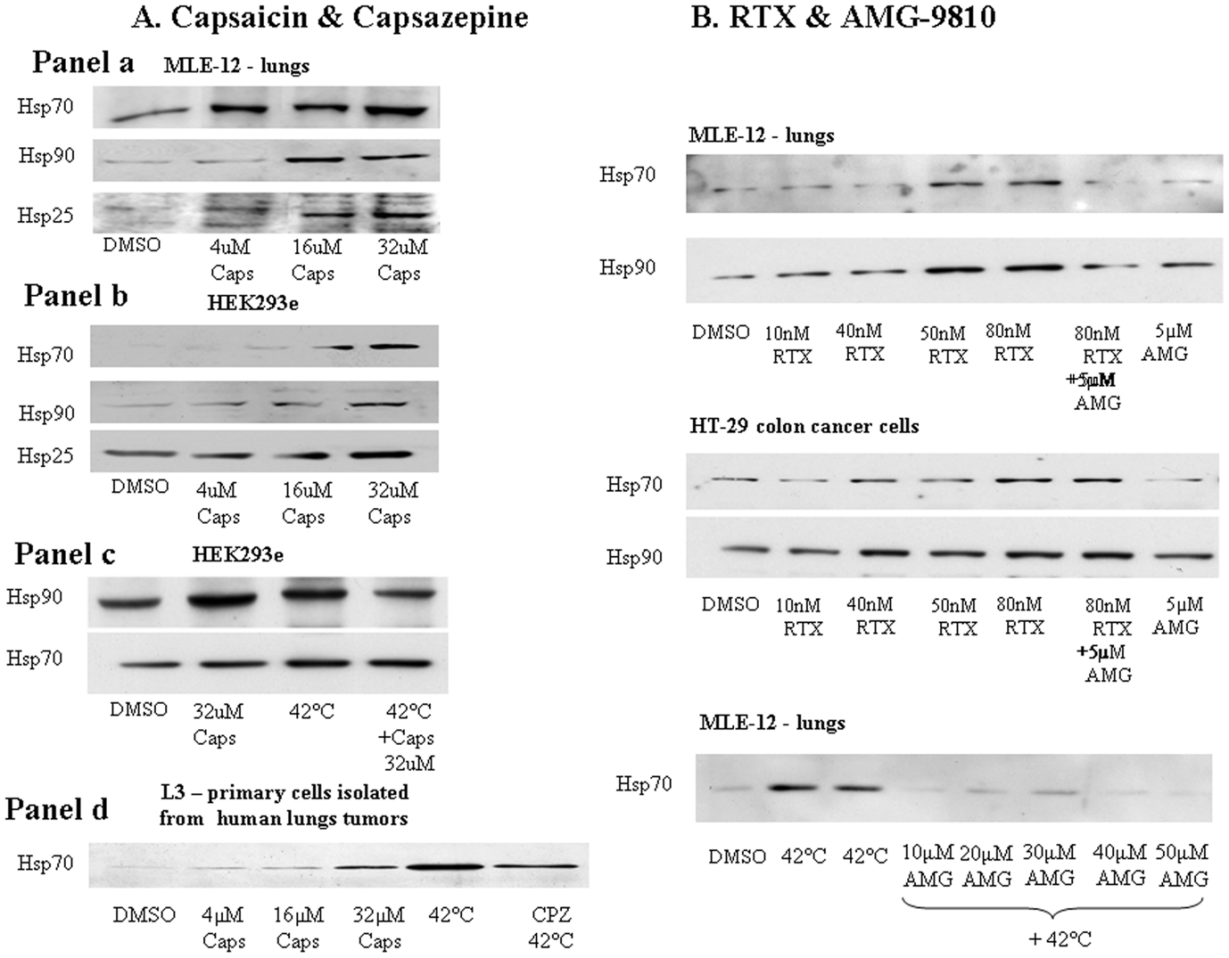
Effect of various TRPV1 agonists and antagonists on heat shock protein expression. A. Capsaicin & Capsazepine: Representative immunoblots analysis of Hsp70, Hsp90 and Hsp27 abundances. Hsp70, Hsp90 and Hsp27 were detected in MLE-12 (Panel a), HEK-293e (Panel b) cells. Hsp70 was detected in Cells in L3 – primary cells isolated from human lung tumors (Panel d). Cells culture were incubated with 0.01% DMSO, 4 µM, 16 µM or 32 µM of capsaicin (Caps) for 1 hr. 15 µg of total protein/lane were separated on 9% SDS-PAGE gel. (Panel c) represents Hsp70 and Hsp90 levels in control HEK-293e cells, HEK-293e cells treated with 32 µM of capsaicin for 1 hr, heat shock treatment for 3 hrs at 42°C and cells treated with both 32 µM of capsaicin for 1 hr and heat shock for 3 hrs at 42°C.B. RTX & AMG-9810: Representative immunoblot analysis of Hsp70, Hsp90. Hsp70, Hsp90 were detected in MLE-12 and HT-29 cells. Cell cultures were incubated with 0.01% DMSO, 10 nM, 40 nM, 50 nM or 80 nM of RTX for 1 hr, 80 nM RTX with the addition of 5 µM of AMG-9810 for 1 hr, 5 µM AMG-9810 at 37°C for 1 hr or 10–50 µM AMG-9810 at 42°C for 3 hrs. 15 µg of total protein/lane were separated on 9% SDS-PAGE gel.

Other TRPV1 agonists and antagonists, RTX and AMG-9810, respectively, were also tested. RTX dose response was clearly associated with increased Hsp90, and Hsp70 expressions, while AMG-9810 blocked these effects (**Fig. 4B**). The RTX findings correlate with the dose response findings of capsaicin (**Fig. 4A**). Doses in the range of 4-32 µM of capsaicin showed elevated levels of Hsp70, Hsp90 and Hsp27 in a dose-dependent manner in epithelial cell cultures (**Fig. 4A**).

### The role of TRPV in the HSR

**Figure 5:**
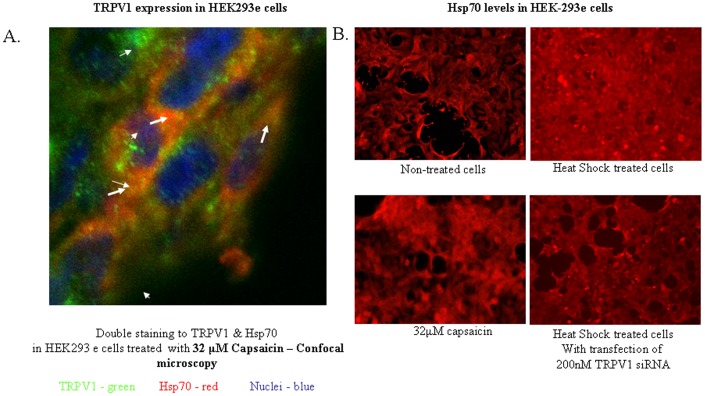
TRPV1 expression in HEK293e cells. A. Left panel: Double immunofluorescence staining of TRPV1 and Hsp70 in HEK-293e cells treated with 32 µM capsaicin for 1 hr. TRPV1 visualized in green, Hsp70 in red and DAPI (Nuclei) in blue. White arrows indicate Hsp70 abundance (left picture) and co-localization (right picture). B. Right panel: Immunofluorescence staining of Hsp70 in HEK-293e cells in non-treated cells, treated cells with 32 µM of capsaicin for 1 hr, heat shock treated cells for 3 hrs at 42C and cells transfected with 200 nM TRPV1 siRNA and treated with heat shock for 3 hrs at 42C.

**Figure 6:**
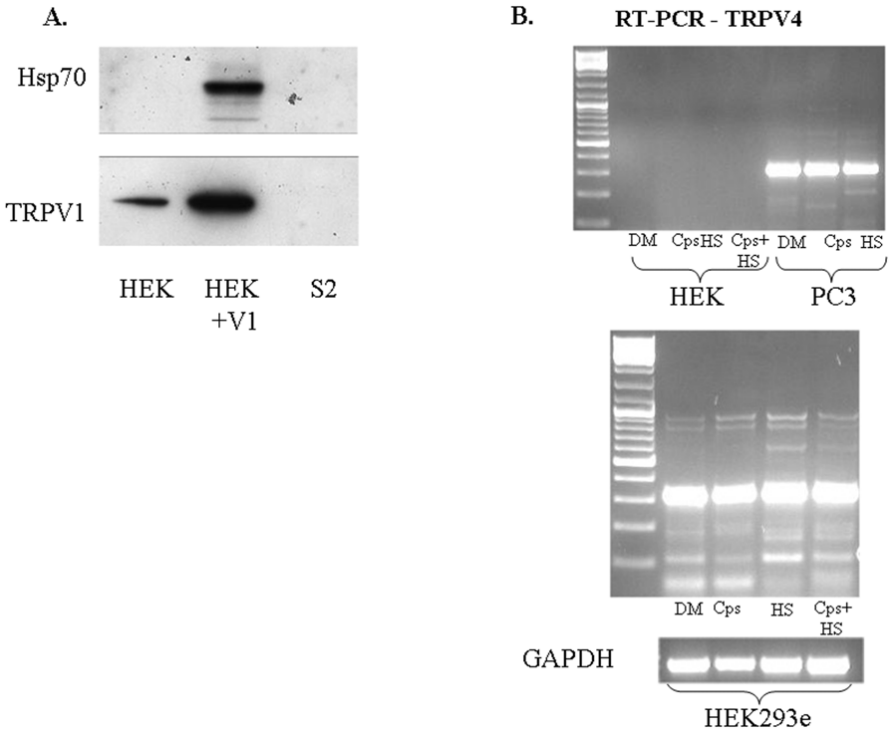
HEK293e cells versus HEK cells and S2 KO cells – TRPV1 & TRPV4 expressions. A. Representative immunoblot analysis of Hsp70 and TRPV1 in HEK with or without the transfection of the TRPV1 gene (HEK, HEK+V1) and S2 drosophila cells lacking the TRPV1 gene (S2). B. Semi-quantitative RT-PCR for TRPV4. Upper panel: mRNA levels from HEK and PC3 prostate cancer cells: non treated cells (DM), cells treated with 32 µM capsaicin for 1 hr (Cps) and cells treated with heat shock at 42°C for 3 hrs (HS) or with heat shock at 42°C for 3 hrs with the addition of capsaicin (Cps+HS). Lower panels: mRNA levels from HEK-293e cells: non treated cells (DM), cells treated with 32 µM capsaicin for 1 hr (Cps), cells treated with heat shock at 42°C for 3 hrs (HS), and cells treated with heat shock at 42°C for 3 hrs with the addition of 32 µM (Cps+HS) and mRNA levels of GAPDH.

In our work, the acute cellular and physiologic effects of heat or capsaicin are principally mediated by the transient receptor potential vanilloid type-1 (TRPV1), a non-selective membrane calcium channel, responsive to noxious temperatures or to vanilloid compounds providing an explanation for the previously unnoted inhibitory effect of EGTA on the heat shock response. The presence of TRPV1 has been previously detected in a wide range of neuronal and non-neuronal cells [31], [32]. In contrast to previous suggestions [33], [34], we found by Western blots the presence of TRPV1 receptors also in HEK-293e cells (**Fig.2B**). A capsaicin dose-response experiment in HEK-293e cells depicted an increased TRPV1 abundance **(** **Fig.2B** **)**. This was also depicted by immuno-histochemistry which showed TRPV1 abundance in capsaicin-treated HEK-293e cells **(** **Fig.5** **)**. To confirm these findings, we performed RT-PCR for TRPV1 (**Fig.2A**). We found TRPV1 expression in both control and heat-treated HEK-293e cells. Interestingly, the mRNA signal was significantly abolished in capsaicin-treated HEK-293e cells in 1 hr. To test these findings, we used prostate cancer cells (PC3), as positive controls expressing TRPV1 [35]. Untreated PC3 cells had lower TRPV1 mRNA levels compared to high levels found following treatment with 32 µM capsaicin for 1 hr, or heat treatment (**Fig. 2A**). This data clearly suggests that TRPV1 is expressed in HEK-293e cells and that presumably, it is over expressed following HS. TRPV1 and Hsp90 protein levels 1 hr after treatment with capsaicin depicted an increased expression compared to control (0 hr) and at 4 and 6 hrs (**Fig. 2C**). Substantiating these findings, a second line of HEK cells devoid of TRPV1 demonstrated that transfection with the TRPV1 gene was associated with an increased expression of Hsp70 in response to treatment with capsaicin (**Fig. 6A**). Furthermore, Drosophila cells, known to be devoid of TRPV1, showed no expression of TRPV1 and no response of Hsp70 to treatment with capsaicin (**Fig. 6A**).

To address the specificity of our finding showing a feedback mechanism causing increased TRPV1 expression following treatment with either capsaicin or heat shock, we performed RT-PCR for TRPV4 depicting a constitutive expression of TRPV4 in HEK293e and PC3 cells following treatment with either DMSO, capsaicin, heat shock or heat shock with capsaicin. There was no effect of these treatments on the level of TRPV4 expression. Furthermore, HEK cells which do not express TRPV1 also do not express TRPV4 (**Fig. 6B**).

**Figure 7:**
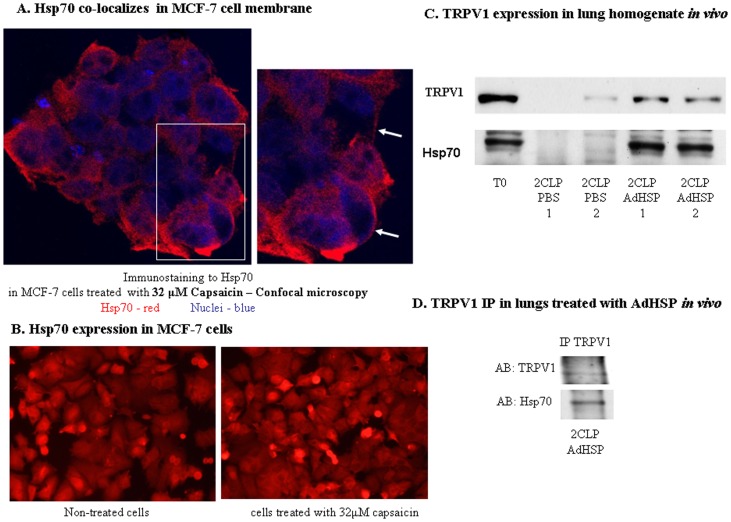
TRPV1 membrane receptor activates the HSR. A. Hsp70 co-localized in MCF-7 cell membrane. Immunofluorescence staining of Hsp70 in MCF-7 cells treated with 32 µM capsaicin for 1 hr. Hsp70 visualized in red and DAPI (Nuclei) in blue. White arrows indicate co-localization of Hsp70 to the membrane (right picture). B. Hsp70 expression in MCF-7 cells. Immunofluorescence staining of Hsp70 in MCF-7 cells in non treated cells compared to treated cells with 32 µM capsaicin for 1 hr. C. TRPV1 expression in lung homogenate *in vivo*. Representative immunoblot analysis of TRPV1 and Hsp70 abundances. TRPV1 and Hsp70 were detected in lungs of untreated rats (T0), septic rats treated with PBS (2CLPPBS) or septic rats treated with adenovirus vector over-expressing Hsp70 (2CLPAdHSP). The numbers (1, 2) represent repeated experiments. D. Co-IPs studies in lung homogenates treated with adenoviral vector over-expressing Hsp70 *in-vivo*. 100 µg of lung homogenates were Co-IPed with TRPV1. Representative immunoblot analysis of TRPV1 and Hsp70 abundances.

These results suggest that the cellular stress response and HSP expression in response to heat treatment is controlled, at least in part, by the membrane-associated TRPV1 receptor, and that capsaicin and RTX can induce an isothermal HSR response in different cell types. In support of this finding, we show a decrease in TRPV1 expression in an *in-vivo* model of sepsis-induced lung injury **(** **Fig.7C**). Moreover, we found that treatment with an adenoviral vector overexpressing Hsp70 maintained high levels of TRPV1 expression in septic lung cells (**Fig. 7C**), suggesting that the protective effects of the HSR is primarily due to the overexpression of Hsp70.

**Figure 8:**
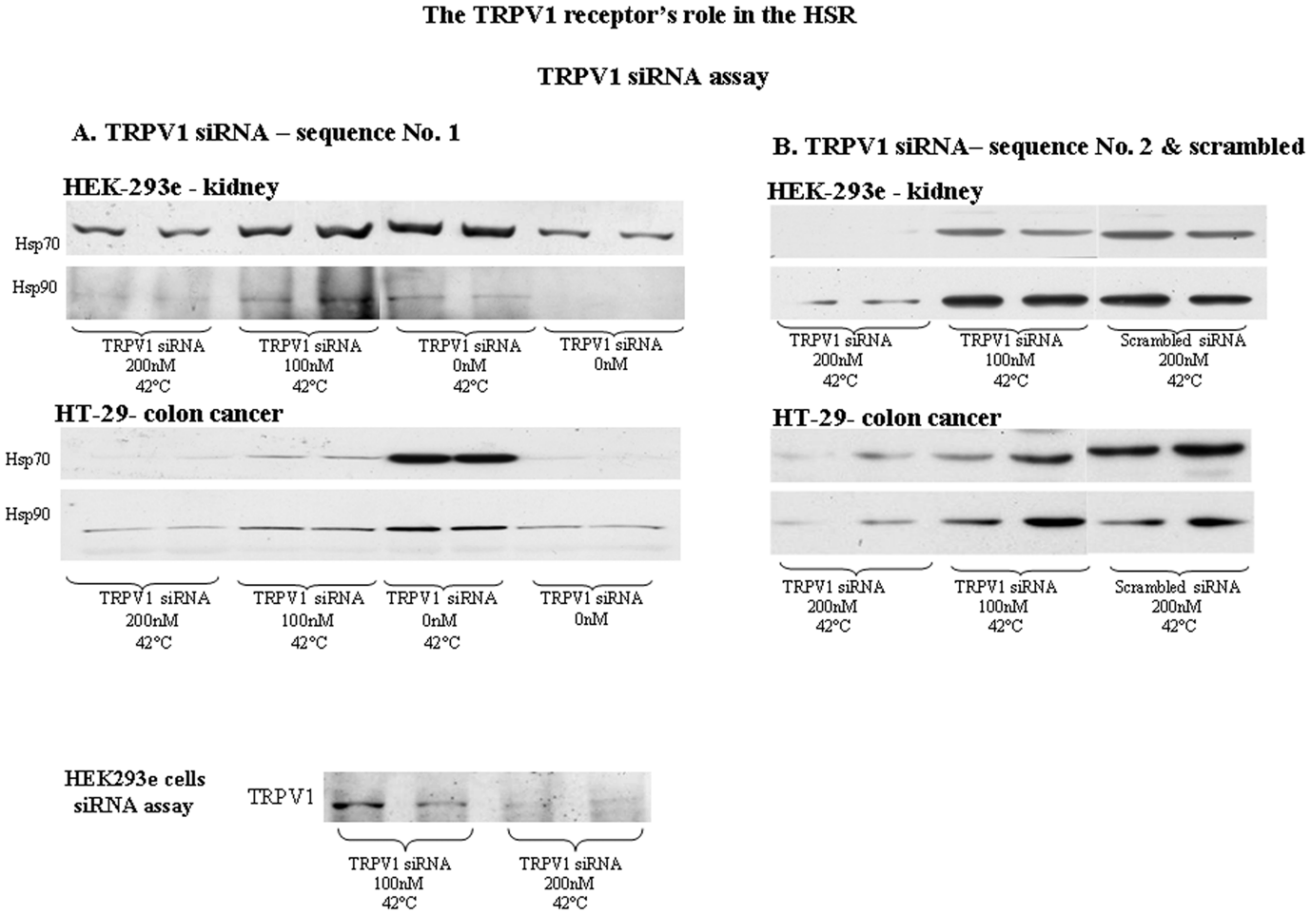
TRPV1 siRNA assay. Upper panels: A. TRPV1 siRNA – sequence No. 1: Knockdown of TRPV1, using siRNA, in HEK-293 and HT-29 cells resulted in inhibition of Hsp90, and Hsp70 expression. HEK293 & HT-29 cells were treated with either oligofectamine alone, heat shock at 42°C for 3 hrs, heat shock at 42°C for 3 hrs with the transfection of 100 nM TRPV1 siRNA or with the transfection of 200 nM TRPV1 siRNA. Cells were transfected with TRPV1 siRNA for 72 hrs, prior to heat shock. Lanes grouped by curly brackets represent repeated RNAi experiments. B. TRPV1 siRNA – sequence No. 2 & scrambled siRNA: Knockdown of TRPV1, using a second sequence of siRNA and 200 nM of scrambled siRNA in HEK-293 and HT-29 cells, resulted in inhibition of Hsp90, and Hsp70 expression.Lower panel: Representative immunoblot of TRPV1 abundance. TRPV1 was detected in HEK-293 cells treated with heat shock at 42°C for 3 hrs with the transfection of either 100 nM TRPV1 siRNA or 200 nM TRPV1 siRNA. Lanes grouped by curly brackets represent repeated RNAi experiments.

Confirming the inhibitory effect by capsazepine, a TRPV1 antagonist, we demonstrate a strong TRPV1-dependence of the HSR using two different sequences of TRPV1 siRNAs. TRPV1 siRNA reduced HSP abundance (**Fig. 8**). Blast analysis showed low homology between TRPV1 siRNA and other TRPVs sequences [33], [36], [37], implying that the observed siRNA effects were specific to TRPV1. Transfection with 200 nM of TRPV1 siRNA attenuated Hsp70 and Hsp90 abundances in a dose-dependent manner, depending on cell type: HEK-293e or HT-29 cells (**Fig. 8**), compared to scrambled siRNA.

### Evidence of Interaction between HSP70 and TRPV1

Co-immunoprecipitation studies in lungs treated, *in-vivo* with an adenoviral vector overexpressing Hsp70 showed an unexpected interaction between TRPV1 and Hsp70 (**Fig. 7D**). Further, *in-vitro* immuno-histochemistry in HEK-293e cells using co-localized staining revealed an association between TRPV1 and Hsp70, preferentially close to the plasma membrane (**Fig. 5A**). Hsp70 levels increased in capsaicin and heat shock treated HEK-293e cells compared to non-treated HEK-293e, and decrease in 200 nM TRPV1 siRNA HEK-293e transfected cells **(** **Fig. 5B** **)**. In cancerous MCF-7 cells, cytoplasmic expression as well as co-localization of Hsp70 to the membrane can be seen **(** **Fig. 7A** **)**.

## Discussion

Here we find that TRPV1 acts as a central component of the cellular thermosensory pathway, with direct effects on HSP expression, and that TRPV1 antagonists can block the cellular HSP expression. It has been previously documented that TRPV1 channels are mostly expressed in primary sensory neurons and that they are naturally activated by heat or by capsaicin, protons, and anandamide [38]. We show evidence that TRPV1 acts as a general cellular stress sensor, which in addition of being a thermosensor, elicits a heat-shock-like cellular stress response following heat or other chemical treatments in epithelial cells, such as in the lungs, as well as in various cancerous cells. We demonstrate that treatment of human embryonic kidney cells, alveolar epithelial cells, human breast cancer cells or colon cancer cells with heat, or TRPV1 agonists, capsaicin or RTX, induced the accumulation of Hsp70, Hsp90 and Hsp27, and Hsp70 and Hsp90, respectively, which are hallmarks of the cellular heat-shock response. Thus, in addition to the well-established ability of capsaicin to induce isothermally the activation of an inter-cellular signal resulting in the deceptive sensation of heat [38], TRPV1 agonists can also induce an intracellular signal resulting in the synthesis of HSPs. Further, we show by immunoblots, RT-PCR and Gel-shift assays that capsaicin treatment resulted in the isothermal activation of HSF-1, suggesting that TRPV1, the only known capsaicin target, is directly responsible for the heat or the chemical signal that up-regulates the heat shock genes, and the cellular heat shock response (HSR) in general. In support of these findings, we found that cells devoid of the TRPV1 gene, could not over express Hsp70 in response to heat-shock or capsaicin treatments. Further, we show that in cells harboring TRPV1, either heat-shock or treatment with capsaicin induces an increase of HSF-1 and Phospho-HSF-1 abundances, a finding confirmed by semi quantitative PCR depicting an increase of HSF-1 mRNA, in agreement with previous reports that riluzole benzothiazole can upregulate mRNA levels of HSF-1 and result in HSP expression [39].

TRPV1 mediates heat- and pain sensations by allowing the transient entry mostly of Ca^2+^, but possibly also Na^+^ and H^+^, into the cytoplasm of neuronal sensory cells [40]. It has been shown that application of capsaicin to HEK-293 cells stably expressing TRPV1, initiates a marked elevation of Ca^2+^ influx [41]. Here, we show that the specific TRPV1 antagonists (capsazepine and AMG-9810) and the non-specific calcium chelator EGTA, blocked heat-induced accumulation of HSPs. Inhibition of Ca^2+^ entry in A-431 cells by removing external Ca^2+^ with the addition of EGTA in the medium greatly attenuated HSF-1 translocation from the cytosol to the nucleus, HSF binding to HSE, Hsp70 gene expression, and protein synthesis [42]. The current model of heat-sensing and signaling in animal cells asserts that the activation of HSF-1 by heat results in the presumed unfolding of some unknown heat-labile proteins in the cell, which would recruit Hsp90, Hsp70 and Hsp40 chaperones away from their normal inhibitory association with HSF-1 [18]. Yet, a different mechanism for heat-sensing and activation by heat or chemicals of the HSR has been initially suggested [22] and was demonstrated in plants [23], whereby activation occurs via an increase in the fluidity of specific membrane domains, leading to the activation of heat-shock genes [22]. Our results support the existence of such a plasma membrane-dependent thermosensory pathway also in mammalian cells, whereby HSF-1 would become activated primarily through subtle changes in the fluidity state of the plasma membrane, which would in turn activate the transient opening of the specific TRPV1 channels, allowing a specific calcium-dependent signal to propagate and activate HSF-1. Furthermore, we and others have shown that membrane fluidizers, such as benzyl alcohol, cause a HSR under non-inducing temperatures in plants and bacteria [43].

The direct heat-sensing or the perception of a non-thermal chemical stimulus by non-neuronal cells as a HSR stimulus, resulting in the accumulation of HSPs may be associated with changes in the membrane state, especially the organization of the membrane micro-domains and the activation of a signal protein such as calmodulin [44]. It has been demonstrated that the C-terminus of TRPV1 contains modulatory domains able to bind calmodulin in the presence of bound Ca^2+^ ions [45]. However, the exact second messengers remain elusive.

Our work extends previous evidence that the primary heat-sensing mechanism activating the HSR is located in the plasma membrane of plants, bacteria, yeast and mammalian cells [46], [47], [48]. Our findings are especially supported by the biochemical, biophysical and pharmacokinetic studies in plants that demonstrated a tight dependence of the heat-induced HSR or isothermal drug treatments, on the transient entry of external Ca^2+^ through specific heat-sensitive Ca^2+^-channels in the plant plasma membrane [49], [50].

Our data point to the plasma membrane-dependent transient potential vanilloid 1 (TRPV) calcium channel-like receptors as being a major heat shock response sensor in different epithelial non-cancerous and cancerous cells, capable of triggering the cellular HSR in response to thermal or isothermal pharmaceutical treatments. Using two different sequences of TRPV1 siRNA, we demonstrated in animal cells dependence between the TRPV1-receptor and the cellular HSR that recapitulated the inhibitory effect of capsazepine. A scrambled siRNA sequence lacked these effects. Cancer cells devoid of TRPV1 were unable to augment a HSR while introducing the TRPV1 into these cells restored this HSR capacity. Furthermore, S2 Drosophila cells, devoid of the TRPV1 gene, treated with capsaicin did not express Hsp70.

TRPV1 is a channel activated by a wide range of agonists (capsaicin, RTX, ethanol, anadamide, NADA, 12-HPETE, camphor, allicin, 2-APB, lidocaine, gingerol, shogaol, piperine monoacylglycerols, w-3 fatty acids, membrane stretch), by low pH and by physical factors such as heat and membrane depolarization [51], [52]. Noxious heat directly gates the channel with a threshold for activation at >43°C. However, as others have noted, the concept of ‘threshold’ is not strictly applicable to a channel such as TRPV1 wherein increases in temperature activate the channel by increasing its probability of activation and wherein the channel, therefore, has some degree of activity at any temperature [53]. Hence, the term ‘threshold’ should be used as an operational definition to indicate the temperature at which the inward current through a thermo-TRP channel becomes large enough to trigger action potentials in afferent nerve fibers. However, here we look at the effect of the TRPV1 channel on the cell itself and conclude that much lower levels of TRPV1 activation may be necessary for the activation of this intra-cellular response.

In all experiments, HSP expression was found to be repeatedly decreased by the exposure to CZP and AMG-9810 at 42°C. However, although capsaicin and RTX are well-documented specific agonists of TRPV1 [54], [28], it cannot be ruled out that some of the depicted effects are also modulated through other non-specific membrane proteins.

Moreover, our data clearly indicate that TRPV1 is not only a sensor of pain and temperature variations that initiate the propagation of a signal among neuronal cells, but it may also act on neuronal as well as non-neuronal cells as a molecular sensor of temperature variation and of other non-thermal cellular stresses, such as inflammatory stimuli (sepsis), activating HSP expression. We demonstrate a significant loss of the TRPV1 receptor in the presence of a septic insult to the lungs and that this loss can be corrected by treatment with an adenovirus expressing Hsp70; this is in agreement with earlier findings indicating that TRPV1 KO mice are highly susceptible to septic insults, compared to the wild type [54]. Further, confocal microscopy in cells treated with capsaicin showed that TRPV1 co-localizes with Hsp70, suggesting that the induction of the HSR promotes the co-localization of Hsp70 to the membrane; this is in accordance with the previously observed case of human tumors, wherein Hsp70 was found to be associated with cholesterol-rich microdomains in the plasma membrane of mouse tumors [55]. Further, our co-immunoprecipitation data suggest a possible interaction between TRPV1 and Hsp70 within lungs that overexpress Hsp70. These changes may be associated with modulation by Hsp70 of TRPV1 trafficking, through PI3K and PKC as shown in sensory nerve endings [55], [56]. Others have shown that Hsp70, together with Hsp90, regulate the function, trafficking and turnover of a wide variety of signaling proteins [57]. Thus, we speculate that overexpression of Hsp70 or induction of the HSR may initiate TRPV1 stabilization or its trafficking to the membrane.

Several tumor cell lines which express high levels of HSPs have been associated with an increased resistance to chemotherapy [58]. Further, the down-regulation of HSP expression or activity resulted in suppressed tumor growth [14], [16]. Here, we demonstrated that capsaicin and capsazepine have similar contradictory effects on cancer cells and on epithelial non-cancerous cells. Others have already described the pro-apoptotic effect of capsazepine [59]. Given that TRPV receptors sense heat and respond to chemicals and to cellular stresses in general, their therapeutic inhibition with specific inhibitors, such as capsazepine, could potentiate various chemotherapies otherwise hindered by an over effective cellular HSR. Furthermore, thermotherapy of particular cancers has been shown to be efficient at first, but a loss of efficacy and resistance was observed, in correlation with increased HSP levels [60]. It is tempting to speculate that by blocking the cellular stress response, capsazepine could rehabilitate cancer thermotherapy [61]. In addition to the possible therapy of resistant cancers associated with HSP over-expression, the pharmacological control of stress-induced HSPs, molecular chaperones in particular, holds promise in the modulation of inflammation, protein misfolding diseases and aging in general.

## Materials and Methods

### Isolation and preparation of cytosolic, nuclear and whole extracts from cells

HEK293e (Human Embryonic Kidney cells found to express TRPV1), HEK cells, a gift from Prof. Baruch Minke's lab, (not expressing TRPV1), S2 (Drosophila) cells a gift from Prof. Baruch Minke's lab, (lack of TRPV1 gene), MLE-12 (Murine Lung Epithelial) cells (obtained from ATCC, Manassas, VA, USA), HT-29 (human colon cancer) cells, PC3 (human prostate cancer) cells and MCF-7 (human breast carcinoma cells) were grown in DMEM or RPMI containing 10% FCS, 100 units/ml penicillin and 100 µg/ml streptomycin (Gibco BRL, Grand Island, NY, USA). Cells were incubated with 0.01% DMSO, 4 µM, 16 µM and 32 µM of capsaicin for 1 hr with or without the addition of 100 µM capsazepine, or treated with either heat shock of 42°C for 3 hrs, with or without 5 mM EGTA (a non-specific calcium chelator) or 100 µM capsazepine. Others have demonstrated that capsaicin concentrations in the order of 10^−4^ M [62] affected neuronal cells in culture. Here, we have used higher capsaicin concentrations in non-neuronal cells, based on dose response curves; these demonstrated that the doses in the range of 4–32 µM did not inadvertently affect these epithelial cell cultures (Data not shown). Additional TRPV1 agonist (RTX) and antagonist (AMG-9810) were added to MLE-12 and HT-29. RTX was added in a dose-dependent manner of 10, 40, 50, 80 nM and AMG-9810 in concentrations of 5-50 µM.

For cell fractionation preparations, media were collected and centrifuged. Detached floating cells (a consequence of addition of capsazepine or EGTA), were added to the attached cell pellets. The cells were washed with PBS x 1 and lysed with lysis buffer containing 10 mM Tris, (pH 7.9), 60 mM KCl, 1 mM EDTA, 1 mM DTT, 0.4% Nonidet P-40, 0.5 mM PMSF, 1 mM Na3-Vo4 and a protease inhibitor cocktail (Roche Diagnostics, GmbH, Manheim, Germany). The lysates were incubated in ice for 10 min and centrifuged at 2,500 rpm for 5 min at 4°C. The supernatants (cytoplasmatic extracts) were collected and pellets were washed three times, suspended in lysis buffer consisting of 20 mM Tris (pH 7.9), 0.4 NaCl, 1.5 mM MgCl2, 1.5 mM EDTA, 1 mM DTT, 25% glycerol and the protease inhibitor cocktails listed above. After suspension and vortex mixing, the extracts were incubated 10 min on ice and centrifuged 5 min at 10,000 rpm. The supernatant (nuclear extract) was collected for nuclear protein detection. The commercial kit (cell lysis buffer by cell signaling, Technology, Inc) was used for whole cell extractions. Protein concentration was determined using the Bradford method.

### Western Blot Analysis

Lysates containing 15 µg of cytosolic, nuclear or whole cell proteins were separated on 9% SDS-PAGE gels. Immunoblot signals were detected using enhanced chemiluminescence and quantified using TINA software with scanning densitometry. Hsp70 was identified with a primary mouse monoclonal Hsp70 antibody (Stressgen, Biotechnologies Corp., Canada), Hsp90 with a primary rat Hsp90 monoclonal antibody (Stressgen, Biotechnologies Corp., Canada), Hsp27 with a primary rabbit polyclonal Hsp27 antibody (Stressgen, Biotechnologies Corp., Canada), HSF-1 with a primary rabbit polyclonal HSF-1 antibody (Santa Cruz Biotech Inc., CA USA), Phospho-HSF-1 with a primary rabbit monoclonal phospho-HSF-1 antibody (Abcam Inc, Cambridge, UK), TRPV-1 with rabbit polyclonal TRPV-1 antibodies (Alomone Labs Inc, Israel and Abcam Inc., Cambridge, UK), β-actin with primary mouse monoclonal β-actin antibody (Abcam Inc, Cambridge, UK). Secondary antibodies were goat anti-rabbit IgG, or goat anti-rat IgG or goat anti mouse IgG (Jackson, Immunoresearch Lab., Inc., West Grove, PA., USA).

### Electrophoretic Mobility Shift Analysis of HSF-1 DNA Binding Activity

A ^32^P-labeled double-stranded DNA oligonucleotide containing a consensus HSE binding site (5′–CTAGAAGCTTCTAGAAGCTTCAG-3′) was used. The labeled oligonucleotide was purified on a G-25 Sephadex column. Nuclear extracts containing 10 µg of protein were incubated with binding buffer (20 mM Hepes, (pH 7.9), 60 mM KCl, 2 mM EDTA, 5 mM MgCl2, 10% glycerol, 1 mM PMSF, 1 mM DTT, 0.1% NP-40), dIdC (1 µg/µl) for 20 min at room temperature. The labeled oligonucleotide was added to the reaction mixture for 20 min. Specificity for the binding site was determined by cold competition using a ten-fold excess of unlabeled oligonucleotide. Complexes were visualized by autoradiography.

### RT-PCR

Total RNAs from the cell tissues were extracted using Trireagent (Sigma, Saint Louis, MO) according to the manufacturer's instructions. 2.5 µg of extracted RNA (for each sample) was reverse transcribed using 0.5 µg/µl oligo dT, 200 u/µl M-MLV RT and 2.5 mM dNTPs. 2 µl of the reacting cDNA products were used as template for PCR. PCR was performed using PCR primers specific for human HSF-1: 5′ –GTGCAGTCAAACCGGATCCT -3′ (sense) and 5′ –GAGATGATGGGTCCAGAGCTG– 3′ (anti sense), and carried out for 40 cycles.

Human TRPV1: 5′ – TGTGCCGTTTCATTTT - 3′ (sense) and 5′ –TGCAGCTTCCAGATGTT - 3′ (anti sense), Human TRPV4: 5′ –CGTGAGAACACCAAGTTTGTT– 3′ (sense) and 5 – CTTGCTGTTGTACACCAGGAT – 3 (anti sense), GAPDH was used as a house keeping control gene, using PCR primers specific for GAPDH: 5′- ACCACAGTCCATGCCATCAC-3′ and 5′- TCCACCACCCTGTTGCTGTA-3′.

### Double immunofluorescence – Confocal microscopy

Double immunofluorescence was performed as follows: cells were fixed in 4% PFA for twenty minutes. Cells were permabilized using PBS×1 buffer containing 5% donkey serum and 0.5% Triton for ten minutes. TRPV1 rabbit polyclonal antibody 1∶200 (Alomone Labs Inc., Israel) and Hsp70 mouse monoclonal 1∶50 (Stressgen, Biotechnologies Corp., Canada) were added for an hour. The secondary antibodies, goat anti rabbit Alexa 488 and goat anti mouse Rodamin X (Jackson, Immunoresearch Lab., Inc., West Grove, PA., USA) were added for thirty minutes. The cells were washed three times and the nuclei were counterstained with DAPI (Vector Laboratories Inc., Burlingame, CA, USA). Confocal imaging was visualized using Nikon Confocal microscope in magnification x 60.

### Induction of Sepsis

All studies were approved by the Institutional Animal Care and Use Committees (IACUC) of both collaborating institutions and conform to both University Laboratory Animal Resources (ULAR), The Hebrew University School of Medicine Ethics Committee – research number MD 109.14-4 and the National Institutes of Health standards. Severe sepsis was induced by cecal ligation and double puncture (2CLP) in male adolescent rats under isoflurane anesthesia as previously described [7]–[9], [63], [64]. Animals were fluid resuscitated (50 cc/kg of 0.9% saline injected subcutaneously) at the time of surgery and at 24 hours. 48 hours after 2CLP, animals were euthanized with an overdose (150 mg/kg) of pentobarbital. Lungs were homogenized for extraction as previously described [7]–[9], [63], [64].

### Adenoviral Vector Administration

As previously described, 10^11^ viral plaque-forming units (PFUs) of AdHSP recombinant E1,E3-deleted adenoviral vector dissolved in PBS (total volume, 300 µl) were administered via tracheal puncture at the time of 2CLP [7]–[9], [63], [64]. Control animals were subjected to 2CLP and treated with intratracheal PBS alone.

### Immunoprecipitation

Immunoprecipitation was performed as previously described [7]–[9]. Briefly, 100 µg of extracts were immunoprecipitated using a rabbit polyclonal anti-TRPV1, (Abcam Inc, Cambridge, UK), that was diluted 1∶100. Samples were agitated overnight at 4°C. Protein A/G beads (Pierce Inc., USA) were added and the samples were agitated for two more hours at 4°C and centrifuged at 14,000 rpm for 5 min at 4°C. The resulting pellet was washed three times, suspended in sample buffer and boiled for 5 min. The samples were centrifuged at 14,000 rpm for 5 min at room temperature and the resulting supernatants were subjected to immunoblot as described above.

### TRPV1 siRNA experiments

RNA interference (siRNA) of human TRPV1 was performed using sequence siRNA reagents (IDT Inc, Coralville, IA, USA) using human TRPV1 siRNA sequence: 5′-GGA TTGCCCTCACGAGGAA-3′ [33]. The second TRPV1 siRNA sequence was: 5′ – AACUGGAGACUAUUUCCGA- 3′ (sense), 5′ – UCGGAAAUAGUCUCCAGUU - 3′ (anti sense). Furthermore, a scrambled siRNA was used as a transfection negative control. HEK293 and HT-29 cells were transfected with TRPV1 siRNA for 72 hrs using Oligofectamine (Invitrogen Inc., California, USA) as recommended by the manufacturer.

### Statistical analysis

Each experiment was repeated at least four times. Where appropriate, a t-test was performed. The significance level was set at *P*<0.05. Bars in the graphs represent the standard error of the mean.
